# Circle of Security (COSP) Implementation in a Public Clinical Setting in Spain

**DOI:** 10.1192/j.eurpsy.2025.839

**Published:** 2025-08-26

**Authors:** L. Garcia Murillo, A. Cañuelo Marquez, L. Mallol, M. Diaz de Neira, M. Morales, I. Palanca

**Affiliations:** 1 Psiquiatría, Hospital Universitario Puerta de Hierro Majadahonda, Majadahonda, Spain

## Abstract

**Introduction:**

Evidence shows that attachment insecurity and disorganization increase the risk of developing psychopathology. The Circle of Security-Parenting Intervention (COSP; Cooper, Hoffman & Powell 2009) is designed to enhance secure attachment between caregivers and children under six years old, based on decades of attachment research. This evidence-based program has been translated into 14 languages and is present in almost 30 countries. However, in Spain, it is not currently offered in the public system, despite having a public health, education, and social services system intended to provide universal coverage.

Our team works in a Child Psychiatry Consultation, where we evaluate toddlers when Autism Spectrum Disorder (ASD) is suspected by general pediatricians or schools. Over the years, we’ve found that many children do not have ASD but instead present attachment difficulties that the system does not currently support.

**Objectives:**

- To provide parents whose children present difficulties that don’t meet the criteria for disorders receiving resources from the educational or social system with tools to help their children.
- To implement an intervention in a public clinical setting in Spain, specifically in a Child Psychiatry Consultation, that could help caregivers with attachment difficulties.

**Methods:**

From the patients referred to our hospital for ASD evaluations, we identified seven parents whose children didn’t meet the criteria for a neurodevelopmental disorder but presented attachment difficulties and could benefit from COSP.

We conducted a group with these parents, consisting of 90-minute sessions over eight weeks. The program provides caregivers with relationship tools, with each chapter building on the previous one. COSP uses visual support and offers participants opportunities to reflect and learn how to better understand and respond to both children’s and caregivers’ needs.

**Results:**

Six of the seven caregivers who began the group completed it. The caregiver who didn’t finish had to leave due to medical issues but requested to rejoin future sessions to complete all eight chapters.

In the satisfaction survey, participants gave the highest possible ratings. Some statements from participants included: “The group has been essential to understanding how I relate to my daughter,” “I can now see relationships from a new perspective,” and “COSP offers very useful tools.”

All participants said they would recommend the program to other parents, stating COSP is something every caregiver should become familiar with.

**Conclusions:**

This pilot group established an initial framework for providing parents who attend our medical consultation and present attachment problems with an intervention that meets their needs.

Given the evidence that secure attachment is key to preventing psychopathology, interventions such as COSP should be more frequently offered.

**Disclosure of Interest:**

None Declared

